# Temporal variability modulates pH impact on larval sea urchin development

**DOI:** 10.1093/conphys/coaa008

**Published:** 2020-04-06

**Authors:** Kit Yu Karen Chan, Chun Sang Daniel Tong

**Affiliations:** Biology Department, Swarthmore College, 500 College Ave, Swarthmore, PA 19081, USA; Division of Life Science, The Hong Kong University of Science and Technology, Clear Water Bay, Kowloon, Hong Kong; Division of Life Science, The Hong Kong University of Science and Technology, Clear Water Bay, Kowloon, Hong Kong

**Keywords:** Biomechanics, ocean acidification, pH fluctuation, pluteus

## Abstract

Coastal organisms reside in highly dynamic habitats. Global climate change is expected to alter not only the mean of the physical conditions experienced but also the frequencies and/or the magnitude of fluctuations of environmental factors. Understanding responses in an ecologically relevant context is essential for formulating management strategies. In particular, there are increasing suggestions that exposure to fluctuations could alleviate the impact of climate change-related stressors by selecting for plasticity that may help acclimatization to future conditions. However, it remains unclear whether the presence of fluctuations alone is sufficient to confer such effects or whether the pattern of the fluctuations matters. Therefore, we investigated the role of frequency and initial conditions of the fluctuations on performance by exposing larval sea urchin *Heliocidaris crassispina* to either constant or fluctuating pH. Reduced pH alone (pH 7.3 vs 8.0) did not affect larval mortality but reduced the growth of larval arms in the static pH treatments. Changes in morphology could affect the swimming mechanics for these small organisms, and geometric morphometric analysis further suggested an overall shape change such that acidified larvae had more U-shaped bodies and shorter arms, which would help maintain stability in moving water. The relative negative impact of lower pH, computed as log response ratio, on larval arm development was smaller when larvae were exposed to pH fluctuations, especially when the change was less frequent (48- vs 24-h cycle). Furthermore, larvae experiencing an initial pH drop, i.e. those where the cycle started at pH 8.0, were more negatively impacted compared with those kept at an initial pH of 7.3 before the cycling started. Our observations suggest that larval responses to climate change stress could not be easily predicted from mean conditions. Instead, to better predict organismal performance in the future ocean, monitoring and investigation of the role of real-time environmental fluctuations along the dispersive pathway is key.

## Introduction

Coastal organisms reside in highly dynamic ecosystems in which physical conditions, such as temperature, salinity, pH and turbulence, vary across multiple spatial and temporal scales (Guadayol *et al.*, 2014; Helmuth *et al.*, 2016). Organisms’ ability to cope with both extremes and fluctuations in these conditions, in turn, shape their population dynamics, and, in the long term, the evolution of traits (Frieder *et al.*, 2014; Evans *et al.*, 2017; Kelly, 2019). Human activities, in particular the emission of carbon dioxide, have altered not only the mean of these physical conditions but also the frequency and magnitude of the variations (Perkins *et al.*, 2012; Hauri *et al.*, 2013; Takeshita *et al.*, 2015; Kwiatkowski and Orr, 2018). Understanding the role of environmental variation in modulating organismal performance is essential for predicting future population and community dynamics and, in turn, for informing sound management strategies (Sorte *et al.*, 2018; Hoshijima *et al.*, 2019).

In coastal systems, pH can vary significantly between tidal cycles, between the day–night cycle due to primary production, between days and weeks due to upwelling or weather and between seasons (Baumann *et al.*, 2015; Kapsenberg and Hofmann, 2016; Evans *et al.*, 2019). Anthropogenic climate change not only reduces mean surface ocean pH but also intensifies pH fluctuations, in particular in coastal habitats (Hauri *et al.*, 2013; Takeshita *et al*., 2015). To better measure the impact of ocean acidification (OA), several studies have assessed the role of diel pH (and oxygen concentration) variation on the early life stages of barnacles, molluscs and fishes, and their responses to OA (Frieder *et al.*, 2014; Eriander *et al.*, 2015; Onitsuka *et al.*, 2018). It is, however, important to note that even if the organisms demonstrate plasticity under present-day fluctuations, the intensity, duration and severity of stressful events could exceed pre-industrial conditions as OA continues to progress (Hauri *et al.*, 2013; Kwiatkowski and Orr, 2018).

Planktonic larvae are often the target of acidification studies as these propagule shape population abundance and distribution through dispersal but are vulnerable to various environmental stresses (Przeslawski *et al.*, 2015; Chan *et al.*, 2018). However, no consistent result has emerged from studies on diel variation. For mussel larvae in the California upwelling system, fluctuating pH reduced the impact of overall pH reduction on early development (Frieder *et al.*, 2014). For the barnacle, *Balanus improvisus*, variation in pH did not affect the mean response but changed the variance in growth and shell mineralogy (Eriander *et al.*, 2015). For fishes, diel pH variation reduced behavioural abnormality of juvenile damselfish*, Acanthochromis polyacanthus*, and clownfish, *Amphiprion percula*, but did not affect the growth of *Amphiprion melanopus* (Jarrold *et al.*, 2017; Jarrold and Munday, 2018). The patterns of pH variations were not directly comparable between these studies. For example, Eriander *et al.* (2015) used a step function change, whereas Frieder *et al.* (2014) compared ambient conditions with minus 0.3 pH unit changes. Thus, it remains unclear which aspect of the fluctuation experienced, e.g. the magnitude, frequency and maximum and minimum value, shaped the responses observed.

This study focuses on the response of the larval sea urchin *Heliocidaris crassispina* to fluctuating pH levels at different time scales. Overall negative responses to OA are well documented for echinoid pluteus larvae (Dupont *et al.*, 2010a; Przeslawski and Byrne, 2013). Typically, metabolic costs (respiration rate, protein turnover and proton pumping) increase with reduced pH for echinoid larvae (Applebaum *et al.*, 2014; Chan *et al.*, 2015; Hu *et al.*, 2018). On the contrary, digestion and clearance rate decrease with pH reduction (Stumpp *et al.*, 2013; Hu *et al.*, 2017). To date, only two published studies have assessed larval urchin responses to fluctuating pH. *In situ* observations of Lamare *et al*. (2016) at a CO_2_ vent found that *Echinometra* embryos had stunted and abnormal development: responses of individuals in this fluctuating environment are similar to those exposed to static conditions in the laboratory. Similarly, exposure to diel variation in pH did not affect larval *Paracentrotus lividus* growth to acidification (García *et al.*, 2018).

Reduction in growth rate of larval body and arms is often reported in OA studies (Dupont *et al.*, 2010b). However, the functional consequences of these morphological changes are little explored. Pluteus larvae use their ciliated arms for both feeding and swimming (Strathmann *et al.*, 2006). Operating in low Reynold’s number environments, changes in size have biomechanical implications for swimming and foraging efficiency (Vogel, 2008). Another consequence of changes in morphology is the altered larval ability to maintain directed upward movement (hereafter referred to as stability). Movement of many passively stable planktonic organisms, e.g. larval sand dollars and algae, subjected to linear shear mimic that of inertial ellipsoids (Durham *et al.*, 2009; Clay *et al.*, 2010; Bearon *et al.*, 2011). When size increases and/or shape changes, fluid exerts an increased torque on the body because the force is applied at points further away from the centre of mass. Meanwhile, the moment of inertia is not significantly increased as the mass remains concentrated at the posterior of the body. This tendency to tilt could, therefore, compromise an organism’s ability to control vertical position in the water column (Chan, 2012). For the pluteus morphology, a hydrodynamic model has suggested that a reduction in the distance between pairs of arms (i.e. an increase in arm elevation angle) could confer stability (Clay *et al.*, 2011). Using geometric morphometric analysis, Chan *et al*. (2011) argued that the observed changes in overall morphology among acidified larval sand dollars were not isometric shrinkage. Instead, larval sand dollars had coordinated shape changes leading to an elevated arm angle when exposed to low pH, which could account for the maintenance of swimming speeds in still water. However, whether these responses in larval echinoids would translate to fluctuating pH exposure is unknown.

The sea urchin *H. crassispina* ranges from the rocky coasts of Japan and Korea to China (Chiu, 1985). This sub-tropical species plays an important role in biogeochemical cycling in coastal habitats through grazing (Wai *et al.*, 2005) and is commercially harvested in South China (Ding *et al.*, 2007). In Hong Kong, this species reproduces from March to October (Urriago *et al.*, 2016). Monthly local marine water monitoring showed that, during the reproductive season, the pH value of surface seawater ranged from pH 7.2 to 8.6 (extreme values from 1986 to 2016; Pecquet *et al.*, 2017). By measuring larval survivorship and growth of sea urchins exposed to static and fluctuating pHs, this study aims to test (i) if variation in rearing pH alleviates the negative impact of low pH; (ii) if experiencing less frequent fluctuation (48-h cycle vs 24-h cycle) reduces larval performance and (iii) if experiencing low pH at the first phase of the cycle negatively affects larval performance.

## Materials and methods

### Adult urchin collection and spawning

Adult sea urchins (*H. crassispina*) were collected by snorkelling near the rocky intertidal outside the Coastal Marine Laboratory at Hong Kong University of Science and Technology (22.33897°N, 114.266815°N). They were kept in a flow-through system at ~ 24°C and salinity of 32 psu and fed *ad libitum* with pre-dried kelp (Laminariaceae) prior to use in the experiment (up to 2 months). Injection of < 0.6 ml of 0.35 M KCl into the coelomic cavity induced urchin spawning (Strathmann, 1987). Sperms were collected dry and kept on ice. Eggs were collected in filtered seawater (FSW, 0.22 μm filtered). The gametes from two males and three females were used in this experiment. The eggs from each female were divided into two beakers and fertilized by the sperm of each male at ~ 1000 sperm ml^−1^. Fertilization success was confirmed by lifting of the fertilization envelope 15 min post-fertilization. After confirming fertilization success, the fertilized eggs were washed with FSW and mixed eggs from the three females were gently pipetted into 2-l rearing jars to achieve a final density of 2 individual ml^−1^.

### Carbonate chemistry manipulation

To assess the effect of pH fluctuations on growth and survival of larval urchins, fertilized eggs were assigned to one of the six treatments. The first two were constant pHs: pH 8.0 as control (Clt) and pH 7.3 representing the average open ocean condition in 2300 and the present-day extreme for Hong Kong (Low; Pecquet *et al.*, 2017). The next two treatments had pH change every 24 h such that the initial conditions (pH 8.0 or pH 7.3) were slowly ramped up or down to the next target pH (pH 7.3 and pH 8.0, respectively) over 6 h (hereafter, Clt2Low_24 and Low2Clt_24). The last two treatments had pH change every 48 h, with the pH level in each jar also altered slowly over 6 h (hereafter, Clt2Low_48 and Low2Clt_48). The experiment ran for a total of 8 days. Each treatment had three replicate jars. All jars were kept at 24.0 ± 0.1°C, salinity of 32 psu, and larvae were fed starting 1 h post-fertilization with *Rhodomonas salina* at 5000 cells ml^−1^. Algae were cultured at ambient pH in f/2 medium and counted with a hemocytometer.

All rearing jars were continuously aerated to provide water mixing through gentle air bubbling. The low pH treatment was achieved by the addition of pure CO_2_ controlled by a mass flow controller (GFC17, AALBORG, USA). The temperature, salinity and total scale pH for each jar were measured every 24 h. pH was measured with a glass electrode (Unitrode, Metrohm, Switzerland) and calibrated with TRIS (Tris/HCl) buffer solution (T31) with a salinity of 33.0 provided by the Dickson Lab at Scripps Oceanographic Institute. Complete water change was performed every other day. Filtered water samples were collected on these days for total alkalinity titration (Metrohm 800 Dosino titrator, Metrohm, Switzerland). Additional samples were also collected on water change days for dissolved inorganic carbon analysis with LiCor Mass Spectrometry (AS-C3, Apollo Technology Solutions LLC, USA). These measurements were benchmarked against standard seawater provided by the Dickson Lab (Batch 140). The carbonate system parameters (*p*CO_2_, Ωa and Ωc) were calculated from these measurements with the R package seacarb (Lavigne *et al.*, 2011) using the dissociation constants from Mehrbach *et al.* (1973) as refitted by Dickson & Millero (1987).

### Larval growth and mortality

**Figure 1:**
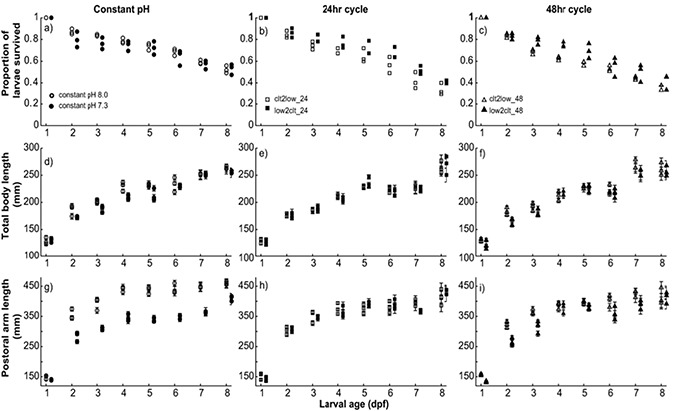
Larval survival decreased under constant pH 8.0, constant pH 7.3 and fluctuating pH between these two levels over 8 days post fertilization (dpf). Each symbol represents the raw count observed within one replicate jar (**a**–**c**). Linear regressions between the observed proportions and time were used to determine the mortality rate (Table S1). Larval growth rate was determined by measuring the total body length (**d**–**f**) and postoral arm length (**g**–**i**) of 15 haphazardly selected individuals from each treatment daily. Means and standard error of means for each replicate jar were plotted for d–i. Logarithmic regressions between the body/arm length and time were used to determine the growth rate (Table S1). Open symbols represent treatments that started with control (pH 8.0) condition, and solid symbols are for those starting with pH 7.3.

Duplicate, 10-ml subsamples were taken from each rearing jar daily. The number of larvae was counted under a dissecting microscope. The change in larval density over time was used to represent larval survivorship. Individuals from the subsamples were preserved with 2% buffered paraformaldehyde. Micrographs were taken for a haphazardly selected subset of the preserved larvae under a compound microscope (H600L, Nikon, Japan) equipped with a digital camera (D5600, Nikon, Japan). The total body length (TBL) and postoral arm length (POL) were measured for 15 individuals from each replicated jar daily (*N* = 2160, Fig. 1) with Fiji ImageJ (Schindelin *et al.*, 2012). Fifteen 8-day-old larvae were selected haphazardly from each treatment for landmark analysis after Chan *et al.* (2011). Coordinates were extracted with tpsDIG2w32 (Rohlf, 2018).

### Statistical analysis

All analyses but the landmark analysis were performed with SPSS 25.0 (IBM, USA) with the significance level set at 0.05. Normality and homogeneity of variance of data were tested with Wilk–Shapiro’s and Levene’s test, respectively. A two-way ANOVA was used to test if the carbonate chemistry varied between date and replicated jars within a treatment. The difference between the two pH levels was confirmed with a *t* test. Linear regression between larval densities and time was performed on individual replicate jars, and the slope of the significant regressions was considered the survivorship (proportion larvae day^−1^). Larval growth rates (both TBL and POL) were determined as the slope of significant logarithmic regression of the lengths and time (μm ln(day)^−1^). The effects of treatments on larval survivorship and growth rates were tested with ANOVAs. To better visualize the impact of pH reduction and fluctuation, log response ratios (LnRR) were computed by comparing the mean of each treatment against that of the control after Hedges *et al.* (1999). The 95% confidence interval for each LnRR was also determined to test if it overlaps with zero. Pairwise comparisons between LnRR were performed with *Z*-tests. A Procrustes analysis was performed on the landmarks collected, a sequent canonical variant analysis was performed to visualize the difference in shape between treatments and the effect of treatment was compared with a Procrustes ANOVA. These geometric morphometric analyses were performed with the software MorphJ (Klingenberg, 2011).

## Results

### Treatment conditions

Overall, the ambient pH for the nominal pH 8.0 treatments was measured as a pH_T_ of 8.00 ± 0.05 (mean ± S.D., (*n* = 36), total alkalinity of 2111 ± 56 μmol kg^−1^ (*n* = 36) and dissolved inorganic carbon of 2013 ± 90 μmol kg^−1^ (*n* = 36). The pH 8.0 treatment corresponded to a *p*CO_2_ level of 445 ± 49 μatm (Table 1). The measured carbonate chemistry parameters were significantly different between those in the nominal pH 8.0 and pH 7.3 treatments (*F*_1, 72_ = 1774, *P* < 0.001). The low pH treatments measured pH_T_ of 7.34 ± 0.02 (*n* = 72), total alkalinity of 2119 ± 48 μmol kg^−1^ (*n* = 36) and dissolved inorganic carbon of 2174 ± 79 μmol kg^−1^ (*n* = 36). These acidified treatments corresponded to a calculated *p*CO_2_ level of 2364 ± 122 μatm and under-saturation of aragonite (Ω_ar_ = 0.7 ± 0.03) but not calcite (Ω_Ca_ = 1.08 ± 0.05).

Although pH varied over time in the constant treatments (*F*_1, 24_ ≥ 21.1, *P* < 0.01), the actual variations were negligible: the range across the duration of the experiment was only 0.06 unit for low pH treatment and 0.11 unit for the control only bubbled with ambient air. The pH between the two target levels was significantly different at all times. In the constant low pH treatment (Low), replicate jars did not have a significant effect on the pH_T_ level (*F*_2, 24_ = 2.22, *P* = 0.146). While there was a statistically significant difference in pH_T_ between replicate jars in the constant pH 8.0 control (*F*_2, 24_ = 2.20, *P* = 0.02), the maximum difference was only 0.03 pH unit on any given day. As expected, there were significant effects of time (days) in the remaining four fluctuating treatments (clt2low_24, low2clt_24, clt2low_48 and low2clt_48; *F*_1, 24_ ≥ 1544.441, *P* < 0.0001). However, no significant difference was detected between replicate jars in these fluctuating treatments (*F*_2, 24_ ≥ 2.039, *P* ≤ 0.167).

### Larval mortality, growth and overall shape

**Figure 2:**
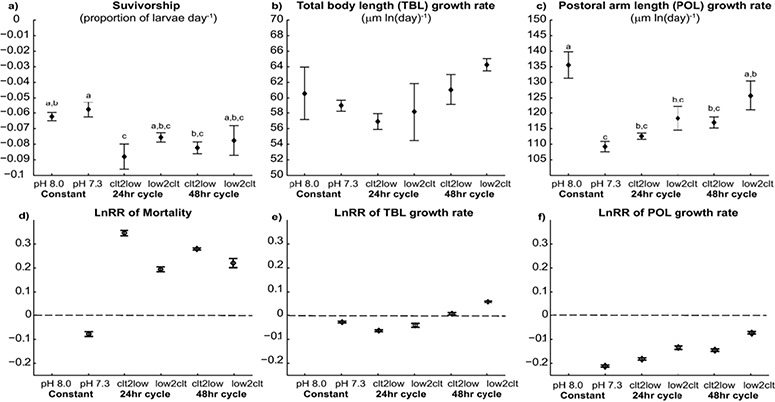
Survivorship (rate of decline in density), total body length and postoral arm length growth rate of larval urchins exposed to constant and fluctuating pH between 8.0 and 7.3. Means and standard errors of means of the three replicate jars per treatment were plotted (*N* = 18, **a**–**c**). Exposure to constant low pH did not significantly increase mortality (pH 7.3 in a) but did compromise the growth rates of arms (pH 7.3 in c). Log response ratio (LnRR) for each treatment relative to the constant pH 8.0 (control) and their corresponding 95% confidence intervals are shown (**d**–**f**). The log response ratio relative to constant pH 8.0 (control) affirmed the pattern observed in the direct measurements i.e. increased larval mortality (d) and a significant reduction in arm growth (f) in the acidified treatments. Pairwise comparisons of the LnRR with *Z*-test showed that the frequency (24- vs 48-h cycle) and the initial condition (clt2low vs low2 clt) can modulate pH impact (d–f). The LnRR and variance of sampling are listed in Table 2.

Larval density, computed as the proportion of larvae remaining from the initial concentration, declined linearly in all replicate jars of all treatments (Fig. 1, Table S1). Larval survivorship, i.e. the slope of these significant regressions, differed significantly between treatments (*F*_5, 18_ = 7.26, *P* = 0.002). Post hoc analysis suggested that larvae in the Clt2low_24 treatment had significantly lower survivorship than those in the Low treatment (Fig. 1). Log response ratio (LnRR) of the mortality rate of acidified treatment compared to the control suggested that four out of the five treatments experienced an increase in mortality relative to the control (Fig. 2).

**Table 1:** Measured carbonate chemistry parameters (pH_T_, TA, DIC) in the experimental treatments (mean and standard errors)

		Measured			Computed			
Treatment	Days	pH_T_	TA (μmol kg^−1^)	DIC (μmol kg^−1^)	Temp (°C)	*p*CO_2_ (μatm)	Ω_ar_	Ω_ca_
Clt	1–8	8.02 ± 0.016	2121 ± 17	1981 ± 21	23.9 ± 0.03	415.2 ± 8.5	2.8 ± 0.03	4.3 ± 0.05
Low	1–8	7.33 ± 0.004	2122 ± 12	2196 ± 13	24.1 ± 0.02	2407.1 ± 33.0	0.7 ± 0.01	1.1 ± 0.01
Clt2low_24	1, 3, 5, 7	7.99 ± 0.012	NA	NA	24.0 ± 0.02	NA	NA	NA
	2, 4, 6, 8	7.34 ± 0.004	2099 ± 15	2167 ± 13	23.9 ± 0.02	2369.0 ± 30.2	0.7 ± 0.01	1.1 ± 0.02
Low2clt_24	1, 3, 5, 7	7.35 ± 0.005	NA	NA	23.9 ± 0.03	NA	NA	NA
	2, 4, 6, 8	7.95 ± 0.004	2125 ± 9	2058 ± 20	23.9 ± 0.05	483.2 ± 6.8	2.5 ± 0.03	3.9 ± 0.04
Clt2low_48	1, 2, 5, 6	8.03 ± 0.007	2060 ± 10	1903 ± 6	23.9 ± 0.03	403.6 ± 7.6	2.7 ± 0.03	4.2 ± 0.05
	3, 4, 7, 8	7.34 ± 0.002	2133 ± 15	2105 ± 36	24.0 ± 0.02	2362.7 ± 32.3	0.7 ± 0.003	1.1 ± 0.04
Low2clt_48	1, 2, 5, 6	7.35 ± 0.003	2120 ± 16	2156 ± 24	24.1 ± 0.01	2347.3 ± 28.8	1.1 ± 0.02	0.7 ± 0.01
	3, 4, 7, 8	7.96 ± 0.008	2116 ± 21	2097 ± 13	24.0 ± 0.03	448.0 ± 12.2	2.7 ± 0.04	4.1 ± 0.07

Salinity of the system was maintained at 32 psu. *p*CO_2_ and carbonate saturation state were computed with CO2SYS. As Ta and DIC samples were only collected every other day, these data were not available for two treatments Clt2low_24 and Low2clt_24 on the odd experiment days.

TBL and POL of larval urchins significantly increased over time in a logarithmic pattern (Fig. 1, Table S1). Treatment alone did not have a significant effect on the growth rate of TBL (*F*_5, 18_ = 1.29, *P* = 0.331, Fig. 2), but had a significant effect on the growth rate of POL (*F*_5, 18_ = 8.917, *P* = 0.001). Post hoc analysis showed that larvae exposed to constant pH 7.3 had significantly shorter POL than those in control. Similarly, the LnRR suggested a slight decrease in the TBL growth rate (~−0.01) and a relatively larger reduction in the POL growth rate (~−0.15) when larvae experience a reduction in pH.

**Table 2:** Log response ratios and their variances of the acidified treatments relative to the constant pH 8.0 control

	Mortality rate		Total body length		Postoral arm length	
			Growth rate			Growth rate	
Treatment	LnRR	Variance	LnRR	Variance	LnRR	Variance
Low	−0.078	0.009	−0.027	0.002	−0.216	0.005
Clt2low_24	0.345	0.010	−0.062	0.002	−0.186	0.005
Low2clt_24	0.194	0.003	−0.041	0.006	−0.136	0.006
Clt2low_48	0.278	0.004	0.008	0.003	−0.148	0.005
Low2clt_48	0.220	0.017	0.059	0.002	−0.076	0.006

Pairwise comparisons between the LnRRs of different treatments with *Z*-tests suggested that larval urchins exposed to constant pH 7.3 had a larger relative increase in mortality (*Z* ≥ 15.7, *P* < 0.001) than those exposed to fluctuating pH (24 and 48-h cycles). Larval urchins under constant low pH also had a larger relative reduction in POL growth rate (*Z* ≤ −4.33, *P* < 0.001). Frequency of the fluctuation affected the LnRRs: larvae experiencing more frequent changes (24-h cycle) had a significantly larger negative change in growth rates (TBL and POL) than those exposed to the 48-h cycle (Z ≤ −5.53, *P* < 0.001). Within the same temporal pattern, initial condition also significantly affect the LnRRs: larvae that were initially exposed to pH 8.0 and subsequently a pH drop had a significantly larger relative increase in mortality (*Z* ≥ −3.39, *P* < 0.001), and relative decrease in growth rate for TBL (*Z* ≤ −20.4, *P* < 0.001) and POL (*Z* ≤ −16.4, *P* < 0.001).

**Figure 3:**
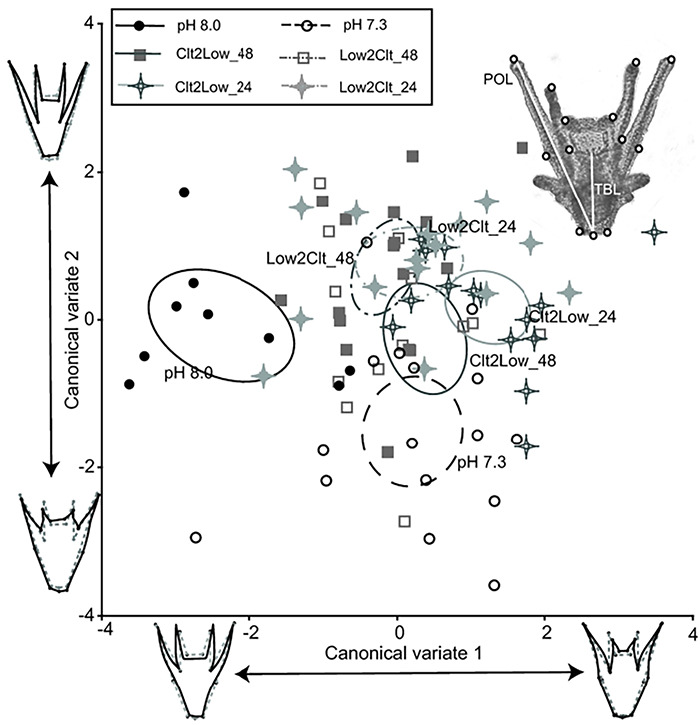
Landmark analysis shows the overall shape of larval sea urchins changed when exposed to acidification stress. Thirteen landmarks were used (circles in the top right inset, total body length (TBL) and postoral arm length (POL) are also labelled). The body shape (V-shape vs. U-shape, CV1) explained 37.6% of the variance, and relative arm length (CV2) explained 27.4% of the variance. Individuals exposed to fluctuations appeared to have an intermediate form. Each data point represents an individual measured (*n* = 15 for each treatment), and the 90% confidence ellipse of the mean is also plotted. Wrapped wireframe (solid line) illustrates the shape change compared to the mean shape (grey, dotted line).

In terms of overall morphology, the different pH treatments had marginally significant effects on both centroid size (*F*_5, 80_ = 2.36, *P* = 0.047) and shape (*F*_110, 1760_ = 1.42, *P* = 0.049) of the subset of 8-day-old larvae measured. Canonical variate analysis (CVA) visualized these observed differences i.e. differences in within-treatment distribution (centroid sizes) and the difference between treatments (shape, Fig. 3). Together, the first two CV accounted for 64.9% of the variance between groups (CV1, eigenvalue = 0.939, % variance = 37.6; CV2, eigenvalue = 0.683, % variance = 27.4). The 90% confidence ellipse of the control (constant pH 8.0) did not overlap with the other five treatments that experienced low pH (Fig. 3). The general morphology is that control larvae had a more V-shaped body (CV1) and relatively longer arms (CV2) than those in the acidified treatments.

## Discussion

Coastal ecosystems are highly variable in space and time. Quantifying the impact of temporal fluctuations could help better predict organismal responses to future climate conditions and therefore inform sound conservation and management strategies. Using the early development of the subtropical sea urchin *H. crassispina* as a model system, we demonstrated that even if the total duration of exposure were identical, the frequency and initial condition of the temporal fluctuations could lead to significant differences in mortality and growth rate. Individuals experiencing temporal fluctuation in pH also exhibited an intermediate shape between the constant low and control pHs. Such a change in morphology would have functional consequences for larval swimming and/or feeding. Our results highlight the need to extend monitoring to better record pH fluctuation and larval distribution patterns in the field in order to improve experimental designs and to aid translation of lab-based observations to the wild.

### Sublethal OA impacts have population-level implications

For most of the echinoid pluteus previously studied, larval mortality was not significantly affected by the reduction of pH within the near-future prediction or within natural variability (Dorey *et al.*, 2013). The reduction in growth, measured through body lengths or arm lengths, is linked to increased metabolic costs, as represented by changes in respiration rate, protein synthesis and digestion rate (Evans *et al.*, 2017, Hu *et al.*, 2018, Lee *et al.*, 2019). The overall acidification impacts observed here are consistent with this earlier work. While there is existing plasticity, it is important to note that, as OA continues to progress, both the mean and extremes of pH and carbonate saturation state experienced by individuals would continue to decrease and could eventually exceed the present-day extremes (Hauri *et al.*, 2013, Takeshita *et al.*, 2015). Long-term, multiple generation experiments are needed to test if such non-genetic variations could be inherited and/or help provide the basis for rapid evolution (Pespeni *et al.*, 2013, Thor *et al.*, 2015, Putnam *et al.*, 2016, Thomsen *et al.*, 2017). Even if only sub-lethal change in larval growth is observed, an increase in pelagic larval duration and the associated mortality could, in turn, reduce larval connectivity between populations (Gerber *et al.*, 2014). These potential climate-driven larval losses have implications for marine reserve designs, e.g. increasing reserve sizes to enhance retention and reducing distance between reserves to promote exchange of individuals (McLeod *et al.*, 2009).

Interestingly, when compared to the congener *Heliocidaris tuberculata*, which experienced a 25% decrease in arm length 72-h post fertilization when reared at pH 7.4 (Byrne *et al.*, 2013), the arm length of *H. crassispina* exposed to pH 7.3 decreased only by 5–7% at 8 days post-fertilization. Hardy and Byrne (2014) suggested that the non-feeding larvae of another congener *H. erythrogramma* are more resilient to acidification than *H. tuberculata* due to relatively larger egg size (>370 μm compared to ~ 90 μm). Interestingly, the average egg size of *H. crassispina* is ~85 μm (*N* = 899, Chan unpublished data). This comparison suggested that in addition to maternal provision, variations in stress tolerance between congeners could be shaped by other environmental and/or biological factors.

### Biomechanical consequences of morphological shifts

The ciliated arms of larval urchins are involved in both swimming and feeding, and thus the observed changes in growth rate of arms and their overall morphologies could affect their swimming speeds, stabilities in moving water and abilities to capture food (Strathmann and Grunbaum, 2006; Chan, 2012). Larvae exposed to constant low pH (7.3) had a more U-shaped body and shorter arms (Fig. 3). This overall morphological change could affect weight distribution, lower the centre of gravity and increase the distance between the centres of gravity and buoyancy, these changes in turn helping to increase the restoring force when an individual is rotated along its vertical axis by shear or turbulence (Grünbaum *et al.*, 2003, Chan *et al*., 2015). Earlier work on sand dollars, *Dendraster excentricus*, exposed to low pH also reported a coordinated change in shape which increases arm elevation angle. Such change in shape was suggested to help maintain stability as the individual was less likely to cut across streamlines in moving water (Chan *et al*., 2011). The current observations on *H. crassispina* reinforce the notion that maintaining swimming capability is essential for pluteus larvae. Interestingly, individuals exposed to fluctuation shared similar scores with the constant pH 7.3 group along CV 1, i.e. more U-shaped body, but they shared similar scores with the constant pH 8.0 group along the CV 2, i.e. longer arms. Further observations are needed to test if the ecological functioning (swimming speed, stability and clearance rate) of these individuals is intermediate between those in constant control and low pHs.

### Fluctuations that modulate OA impact highlight the need for monitoring

Our observations agree with previous observations on bivalves and fishes that the presence of fluctuation could ameliorate acidification stress (Frieder *et al.*, 2014, Gobler *et al.*, 2017, Jarrold *et al.*, 2017, Jarrold and Munday, 2018). Larval *H. crassispina* exposed to fluctuation between pH 7.3 and pH 8.0 had relatively lower mortality and faster arm growth compared to those held at constant pH 7.3 (Fig. 2). This comparison suggested that the stress response was not determined by the minimum pH level experienced. Earlier work on another urchin species *Paracentrotus lividus* suggests that diel fluctuation from pH 8.1 to 7.7 had no significant effect on the growth rate of arms (García *et al.*, 2018). These contrasting outcomes could be attributed to the differences in experimental design, especially in terms of the magnitude of the pH change. Kapsenberg *et al.* (2018) showed that the percent of individuals showing abnormal development increases with increasing amplitude of the pH fluctuation in larval mussels. Alternatively, the difference reflects species-specific buffering capacity and/or ion regulation (Catarino *et al.*, 2012).

Not only does our work show that the presence of temporal fluctuation could shape the developmental response of urchins to acidification stress; we also highlighted that the pattern of fluctuation matters. *In situ* observations of coastal habitats suggest that pH fluctuations similar to the ones used in our experiments are present at the scale of days. Often, these multi-day fluctuations are of a magnitude larger than those observed in a diel cycle (Hofmann *et al.*, 2011, Yu *et al.*, 2011, Kapsenberg and Hofmann, 2016). Similar to mussel larvae, for which exposure to pH fluctuations elevated metabolic cost (Mangan *et al.*, 2017), we observed that more frequent pH fluctuations (24 vs 48-h cycle) are more detrimental to larval survival and growth. These observations highlight the importance of long-term and continuous monitoring of carbonate chemistry changes in order to better assess biological and socio-economic impacts.

However, it is unclear whether such results can be directly applied to repeated exposure to sub-lethal acidification stress in the field. Acclimation to temperature, salinity and pH stress have been reported in adult urchins (Roller *et al.*, 1993, Uthicke *et al.*, 2013, Suckling *et al.*, 2015, Brothers *et al.*, 2016). Such stress tolerances in acclimated adult echinoderms have been suggested to be passed onto their offspring (Dupont *et al.*, 2013, Pespeni *et al.*, 2013, Hu *et al.*, 2018). If heritable acclimation occurs, high-frequency pH fluctuations could potentially act as a selective filter (Sunday *et al.*, 2014). Nevertheless, a survival-reproduction trade-off has been reported in *Drosophila melanogaster* exposed to repeated, sub-lethal stress (Marshall *et al.*, 2010). To better predict physiological responses in the wild, further investigations into the acclimation to repeated pH stress, the possibility of facilitation towards acclimating to other abiotic stress, and trade-offs (e.g. larval vs. post-settlement survival) are needed.

Our results also suggested that larvae that experienced an initial pH drop (Clt2Low_24 and Clt2Low_48) were more negatively impacted in terms of survival and growth than those that experience a pH increase (Low2Clt_24 and Low2Clt_48). Sensitivity to pH and other environmental stress, e.g. warming, has been reported to be stage-dependent in sea urchins (Dupont *et al*., 2010b, Przeslawski and Byrne, 2013, Collin *et al.*, 2016). Given *H. crassispina* is a tropical species with fast development (Onoda, 1931), larval energy allocation might have shifted during the first 48 h of larval development. Differential OA responses observed between pre-feeding and feeding stages have been reported in other urchins. Acidified purple urchins increased the relative allocation of total ATP to protein synthesis and ion transport from 47% on Day 2 to 81% when feeding was initiated on Day 6 (Pan *et al.*, 2015). Lee *et al.* (2019) also observed a reduction in oxygen consumption for larval purple urchins exposed to pH 7.0 during the pre-feeding stage, whereas, in contrast, respiration rate was three times higher at pH 7.0 than at pH 8.2 for feeding larvae. Given that the pre-oral ciliated band develops in front of the mouth of *H. crassispina* within the first 48 h (Thet *et al.*, 2004), the observed difference in sensitivity between larvae experiencing an initial pH drop versus those that experience an increase highlights the need to perform finer temporal observations to test the hypothesis that initiation of feeding elevates sensitivity to acidification stress.

Early development of the sea urchin *H. crassispina* is robust to reduced pH as low as pH 7.3. Larvae exposed to fluctuating pH were less impacted compared to those exposed to constant low pH, suggesting that mean pH and not the minimum pH experienced is a better predictor of larval response to acidification. Temporal variability (frequency and initial condition) of the fluctuation further modulates the impact, highlighting the importance of quantifying repeated acute stress exposure and stage-dependent stress responses. To better predict larval performance under near-future conditions, we need to better quantify variations in environmental conditions along the dispersive pathways and measure how larvae integrate and respond to this information.

## Supplementary Material

Supplementary_Table_S1_coaa008
