# Supplementary material for: Temporal variability modulates pH impact on larval sea urchin development
Source: Conserv Physiol. 2020 Apr 6;8(1):coaa008. doi: 10.1093/conphys/coaa008 (PMC7132065; doi:10.1093/conphys/coaa008)
Supplement: Supplementary_Table_S1_coaa008 [file supplementary_table_s1_coaa008.docx]

**Table S1.**

Mortality rate, total body length (TBL), and postoral arm length (POL) growth rate of larval urchins exposed to static and fluctuating pH. Mortality rate was computed as change in density (individual ml^-1^) over time through linear regression. Growth rates were determined with logarithmic regressions.

|  |  | Equation | R^2^ | F | *p* |
| --- | --- | --- | --- | --- | --- |
| Mortality Rate | | | | | |
| Control | 1 | % survived = -0.06(day)+1.03 | .97 | 214.88 | <0.0001 |
|  | 2 | % survived = -0.06(day)+1.02 | .98 | 261.67 | <0.0001 |
|  | 3 | % survived = -0.07(day)+1.05 | .96 | 150.39 | <0.0001 |
| Low | 1 | % survived = -0.06(day)+1.03 | .95 | 123.26 | <0.0001 |
|  | 2 | % survived = -0.05(day)+0.96 | .87 | 41.33 | <0.0001 |
|  | 3 | % survived = -0.06(day)+0.95 | .86 | 36.89 | 0.001 |
| Clt2Low_24 | 1 | % survived = -0.10(day)+1.06 | .99 | 503.54 | 0.001 |
|  | 2 | % survived = -0.09(day)+1.03 | .96 | 151.88 | <0.0001 |
|  | 3 | % survived = -0.08day)+1.05 | .96 | 152.40 | <0.0001 |
| Low2Clt_24 | 1 | % survived = -0.07(day)+1.09 | .92 | 64.76 | <0.0001 |
|  | 2 | % survived = -0.07(day)+1.03 | .96 | 135.79 | <0.0001 |
|  | 3 | % survived = -0.08(day)+1.07 | .98 | 236.16 | <0.0001 |
| Clt2Low_48 | 1 | % survived = -0.08(day)+1.02 | .96 | 156.60 | <0.0001 |
|  | 2 | % survived = -0.08(day)+0.98 | .92 | 70.89 | <0.0001 |
|  | 3 | % survived = -0.09(day)+1.01 | .96 | 135.67 | <0.0001 |
| Low2Clt_28 | 1 | % survived = -0.07(day)+1.03 | .94 | 89.33 | <0.0001 |
|  | 2 | % survived = -0.09(day)+1.05 | .96 | 157.82 | <0.0001 |
|  | 3 | % survived = -0.07(day)+1.04 | .99 | 426.88 | <0.0001 |
| TBL growth rate | | | | | |
| Control | 1 | TBL = 59.5 (ln (day) )+139.3 | .72 | 306.70 | <0.0001 |
|  | 2 | TBL = 66.0 (ln (day) )+126.9 | .83 | 585.25 | <0.0001 |
|  | 3 | TBL = 55.4 (ln (day) )+141.1 | .77 | 387.92 | <0.0001 |
| Low | 1 | TBL = 58.4 (ln (day) )+128.8 | .83 | 593.32 | <0.0001 |
|  | 2 | TBL = 58.1 (ln (day) )+130.7 | .79 | 446.23 | <0.0001 |
|  | 3 | TBL = 60.4 (ln (day) )+125.0 | .83 | 583.28 | <0.0001 |
|  |  |  |  |  |  |
| Clt2Low_24 | 1 | TBL = 56.5 (ln (day) )+129.2 | .76 | 383.33 | <0.0001 |
|  | 2 | TBL = 58.8 (ln (day) )+131.3 | .66 | 225.39 | <0.0001 |
|  | 3 | TBL = 55.4 (ln (day) )+131.8 | .74 | 335.43 | <0.0001 |
| Low2Clt_24 | 1 | TBL = 53.7 (ln (day) )+131.7 | .67 | 238.35 | <0.0001 |
|  | 2 | TBL = 55.3 (ln (day) )+132.6 | .70 | 270.92 | <0.0001 |
|  | 3 | TBL = 65.5 (ln (day) )+122.5 | .75 | 354.90 | <0.0001 |
| Clt2Low_48 | 1 | TBL = 64.3 (ln (day) )+127.0 | .78 | 407.30 | <0.0001 |
|  | 2 | TBL = 61.4 (ln (day) )+131.7 | .78 | 423.76 | <0.0001 |
|  | 3 | TBL = 57.5 (ln (day) )+126.2 | .70 | 280.42 | <0.0001 |
| Low2Clt_28 | 1 | TBL = 65.8 (ln (day) )+117.0 | .76 | 371.22 | <0.0001 |
|  | 2 | TBL = 63.8 (ln (day) )+125.5 | .76 | 365.53 | <0.0001 |
|  | 3 | TBL = 63.2 (ln (day) )+116.1 | .75 | 357.01 | <0.0001 |

| POL growth rate | | | | | |
| --- | --- | --- | --- | --- | --- |
| Control | 1 | POL = 135.8 (ln (day) )+ 206.4 | .72 | 309.25 | <0.0001 |
|  | 2 | POL = 140.9 (ln (day) )+ 204.3 | .78 | 416.40 | <0.0001 |
|  | 3 | POL = 127.2 (ln (day) )+221.5 | .70 | 278.13 | <0.0001 |
| Low | 1 | POL = 108.5 (ln (day) )+174.0 | .79 | 436.13 | <0.0001 |
|  | 2 | POL = 106.7 (ln (day) )+179.2 | .78 | 411.03 | <0.0001 |
|  | 3 | POL = 112.4 (ln (day) )+165.5 | .81 | 490.18 | <0.0001 |
| Clt2Low_24 | 1 | POL = 112.8 (ln (day) )+180.2 | .74 | 343.72 | <0.0001 |
|  | 2 | POL = 114.0 (ln (day) )+ 200.1 | .71 | 288.07 | <0.0001 |
|  | 3 | POL = 110.7 (ln (day) )+ 203.5 | .72 | 305.02 | <0.0001 |
| Low2Clt_24 | 1 | POL = 118.9 (ln (day) )+183.9 | .74 | 337.90 | <0.0001 |
|  | 2 | POL = 114.4 (ln (day) )+190.0 | .67 | 238.25 | <0.0001 |
|  | 3 | POL = 124.6 (ln (day) )+184.1 | .74 | 330.04 | <0.0001 |
| Clt2Low_48 | 1 | POL =120.5 (ln (day) )+ 198.7 | .69 | 267.84 | <0.0001 |
|  | 2 | POL = 114.8 (ln (day) )+206.9 | .71 | 293.27 | <0.0001 |
|  | 3 | POL = 115.5 (ln (day) )+206.5 | .68 | 255.59 | <0.0001 |
| Low2Clt_28 | 1 | POL = 116.9 (ln (day) )+160.7 | .73 | 322.07 | <0.0001 |
|  | 2 | POL = 170.1 (ln (day) )+127.0 | .72 | 302.91 | <0.0001 |
|  | 3 | POL = 157.5 (ln (day) )+133.1 | .71 | 286.79 | <0.0001 |
